# Dry Coating with Hydrophilic and Hydrophobized Nanostructured Fumed Alumina (Al_2_O_3_) on SiO_*x*_/C Anodes for Enhanced Lithium‐Ion Battery Performance

**DOI:** 10.1002/open.202500170

**Published:** 2025-04-21

**Authors:** Ana L. Azevedo Costa, Daniel Esken, Tatiana Gambaryan‐Roisman, Frank Menzel

**Affiliations:** ^1^ Smart Materials Evonik Operations GmbH Rodenbacher Chaussee 4 63457 Hanau‐Wolfgang Germany; ^2^ Institute for Technical Thermodynamics Technical University of Darmstadt Peter‐Grünberg‐Str. 10 64287 Darmstadt Germany

**Keywords:** dry particle coatings, electrochemistry, lithium‐ion batteries, materials science, silicon‐based anodes, surface coatings

## Abstract

Silicon‐based anode materials hold great promise for advancing lithium‐ion battery technology due to their high specific capacity, low voltage platform, abundant resources, and environmental benefits. However, their inherent challenges, such as poor electrical conductivity, significant volume expansion, and instability of the solid‐electrolyte interphase layer, hinder their widespread commercialization. This study addresses these issues using the dry particle coating method with nanostructured fumed aluminum oxide (Al_2_O_3_), a novel approach with significant potential for commercial scalability. The impact of surface wettability on performance is studied by applying metal oxide coatings, using hydrophilic and hydrophobized surfaces. Electrochemical evaluation shows a significant increase in rate performance and cycle life when the surface coating is applied, with improvements in discharge capacity of around 10% and 17% for hydrophobized and hydrophilic Al_2_O_3_ coatings, respectively, after 100 cycles. The Al_2_O_3_ coating protects the surface of the active material, preventing particle pulverization, reducing side reactions, and decreasing electrolyte decomposition and hydrofluoric acid content. While overall performance improves with coating, the best results are achieved with the hydrophilic coating, which fosters a more homogeneous microstructured electrode. These findings underscore the potential of the dry particle coating technique with Al_2_O_3_ to enhance Si‐based anode performance and facilitate commercial application.

## Graphical abstract

This research explores dry particle coating as an efficient approach to enhance silicon‐based anodes using nanostructured fumed alumina. Herein, it is investigated how coating wettability impacts morphology, composition, and electrochemical performance. Results demonstrate significant improvements in rate capability and cycle life, especially with hydrophilic coatings, which effectively mitigate silicon pulverization and detrimental side reactions.

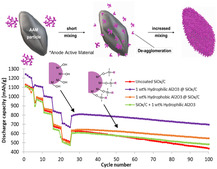

## 1 Introduction

Currently, LIBs are considered the most promising energy storage system due to high energy and power density, long service life, low self‐discharge rate, and environmental benignity compared to other commercial systems.^[^ ^1^, ^2^, ^3^ ^]^ However, the rapidly increasing demands of the growing population and industry impose the requirement to develop LIBs with even higher energy and power densities.

Graphite is dominantly used as anode active material in current commercial LIBs due to its high electronic conductivity, high first cycle coulombic efficiency (FCE, 90–95%) and cycling CE (CCE, >99.9%), and relatively low cost.^[^ ^4^, ^5^, ^6^, ^7^ ^]^ However, its low energy density (≈150 Wh kg^−1^) and theoretical capacity (372 mAh g^−1^), due to the structure accommodating only one lithium ion per six carbons, limit the potential for higher energy/power densities and longer cycle life.^[^ ^8^, ^9^ ^]^ Unlike graphite, many other electrode materials, including silicon (Si), react through alloying with lithium, allowing for much higher capacities.^[^ ^10^, ^11^, ^12^, ^13^, ^14^ ^]^ Si is particularly promising, due to its high theoretical capacity of around 4200 mAh g^−1^, which is more than ten times that of graphite.^[^ ^10^, ^11^, ^12^, ^15^, ^16^ ^]^

However, the successful commercialization of Si‐based anodes faces significant challenges, such as substantial volume changes (around 300% in the lithiated state), which can destroy the solid‐electrolyte interphase (SEI) between the Si anode and electrolyte, causing cracks and pulverization. The cracks can electrochemically isolate particles, facilitating continuous SEI formation and leading to capacity fading and limited lifecycle.^[^ ^17^, ^18^ ^]^ Additionally, the intrinsic low electronic conductivity and low lithium diffusion rates of Si hinder the rate capability of the electrode.^[^ ^19^, ^20^ ^]^ Other sources of instability are the chemical and electrochemical reactivity of Si and the SEI with the LiPF_6_^−^ carbonate electrolyte.^[^ ^21^, ^22^, ^23^ ^]^ At low potentials (≈0.01–1.00 V vs Li|Li^+^), carbonate‐based electrolytes are outside their electrochemical stability window, leading to SEI formation and irreversible Li^+^ consumption in the cell as compounds such as LiF, lithium oxide (Li_2_O), and Li carbonates form.^[^ ^24^, ^25^, ^26^ ^]^

These challenges significantly impede the cyclability of Si‐based electrodes, preventing them from meeting commercial battery system requirements. To address volume expansion and conductivity issues, Si@C composites are designed, leveraging carbon's good electrical conductivity and stress‐buffering nature to form a stable SEI on Si surfaces.^[^ ^27^, ^28^ ^]^ SiO_*x*_ materials also suffer from low initial coulombic efficiency (ICE), consuming lithium ions and reducing energy density. In full‐cell batteries, the limited Li^+^ supply from the cathode and its continuous consumption cause capacity loss prelithiation can be adopted to improve CE and cyclability by compensating for lithium loss during initial cycles.^[^ ^29^ ^]^

Hence, the chosen anode active material for this work is a prelithiated SiO_*x*_ core with a carbon shell.

Furthermore, surface coatings have emerged as an effective approach to mitigate both mechanical and chemical degradation of electrodes. Surface coatings serve to suppress side reactions during battery operation, improve structural integrity during cycling, and enhance ion/electron conductivity for better rate performance.^[^ ^30^, ^31^ ^]^ Electron‐resistive/insulating coatings decrease electrolyte reduction and lithium loss associated with SEI formation.^[^ ^32^ ^]^ Transition‐metal oxides such as Al_2_O_3_, TiO_2_, and MgO have been shown to improve LIB electrode performance, especially for Si‐based anodes.^[^ ^33^ ^]^ Al_2_O_3_, in particular, can enhance electrochemical performance by improving mechanical integrity and reducing electrolyte decomposition.^[^ ^13^, ^14^, ^32^, ^34^, ^35^, ^36^, ^37^, ^38^, ^39^, ^40^, ^41^ ^]^ While chemical methods like chemical vapour deposition and solgel, and physical methods like atomic layer deposition are common coating techniques, wet coating methods pose environmental concerns due to waste streams and volatile organic compounds emissions.^[^ ^30^, ^42^ ^]^ Dry coating, a promising solvent‐free alternative, uses mechanical forces to attach guest nanoparticles onto micron‐sized host particles, relying on van der Waals interactions.^[^ ^42^, ^43^ ^]^ Compared to conventional dry coating approaches, which often require additional processing steps such as heat treatments or aggressive milling, our method uniquely applies nanostructured fumed Al_2_O_3_ directly onto SiO_*x*_/C without further treatment. This simplified approach not only maintains the structural integrity of the active material but also minimizes energy consumption and processing costs, making it a more scalable and environmentally friendly alternative.

In this article, we present a promising method to enhance battery performance by dry coating SiO_*x*_/C anode active material with nanostructured fumed Al_2_O_3_, using both hydrophilic and hydrophobized surfaces without further treatment. The coating improves interaction with the electrolyte, reducing degradation and HF production, and acts as a protective layer for the anode material. This approach significantly enhances cycling stability, rate performance, and capacity retention, using only 1 wt% of inactive material. Additionally, the hydrophilic coating ensures a more homogeneous distribution of active material within the slurry and the finished electrode, resulting in significantly improved overall performance.

## 2 Experimental Section

### 2.1 Dry Coating Process

The purpose of the dry particle coating is to adhere uniformly fine “guest” particles (in size of 0.1–50 μm)^[^ ^44^, ^45^ ^]^ to the surface of a large “host” particle (in size of 1–500 μm),^[^ ^44^, ^45^ ^]^ employing only strong adhesion forces.^[^ ^43^, ^46^, ^47^, ^48^ ^]^ A high number of collisions between the particles occur, and the dominant van der Waals forces create a strong cohesive bond between the host and the guest particles, ultimately resulting in a coating of the guest on the surface of the host particles. This technology's scalability and ease of implementation make it a promising solution for large‐scale commercial applications.^[^ ^42^, ^48^ ^]^ Its solvent‐free nature and lack of additional drying steps contribute to lower production costs and a reduced environmental footprint, making it an attractive alternative for widespread use.^[^ ^43^, ^46^, ^47^, ^48^, ^49^, ^50^, ^51^ ^]^

**Figure 1:**
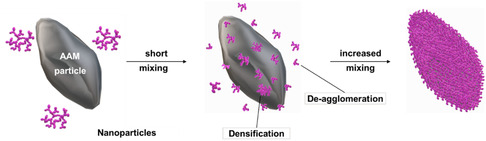
Schematic drawing of the dry coating process.

In this study, micron‐sized SiO_*x*_/C particles act as host particles and nanostructured fumed Al_2_O_3_ as guest particles. The Al_2_O_3_ nanoparticles were produced by flame hydrolysis, which inherently means the formation of nanoparticle aggregates with very strong chemical and sintering bonds.^[^ ^52^ ^]^ Agglomeration is a common phenomenon for fine particles due to van der Waals’ forces.^[^ ^46^ ^]^ Therefore, during the high‐energy mixing process, the device must supply sufficient mechanical force to efficiently promote the deagglomeration of the Al_2_O_3_, while ensuring the structure of the anode active material is not damaged. This guarantees the guest particles are sufficiently small that the van der Waals forces are strong enough to keep them attached to the host particle and thus deliver a strong adhesive coating layer.^[^ ^42^ ^]^ The adhesion force between the smaller particle and the larger particle is greater than the weight of the smaller particle, and hence it is not easily removed from the host. This phenomenon is usually referred to as ordered mixing.^[^ ^42^, ^46^ ^]^ **Figure** **1** shows a schematic representation of this process.

The achieved coating consists of 3D nanostructured different‐sized aggregates, rather than a homogeneous layer or spherical nanoparticles. Depending on operating conditions (processing time, weight fraction of guest to host particles, and particle properties), either a discrete or continuous coating of guest particles can be achieved. While continuous coatings are generally preferred, discrete coatings are ideal when the goal is to change a specific surface property, but a complete shielding of the underlying core particle is undesirable.^[^ ^42^ ^]^

The thickness and uniformity of the Al_2_O_3_ coating layer can be tuned primarily by adjusting three key parameters of the dry coating process: the weight ratio of guest particles to host particles, the mixing intensity, and the mixing duration. First, increasing the Al_2_O_3_ wt% relative to SiO_*x*_/C naturally raises the potential coating thickness because more guest material is available to form a continuous layer on the host surface. Second, higher mixing speeds increase deagglomeration of the Al_2_O_3_ aggregates, leading to more uniform dispersion of smaller Al_2_O_3_ particles that may create thinner but more homogeneous coatings. In contrast, lower speeds may result in larger Al_2_O_3_ clusters adhering to the host, producing thicker regions. Third, extending the mixing time can further promote deagglomeration and allow more Al_2_O_3_ particles to adhere to any uncovered surface areas, potentially increasing the thickness of the coating.

### 2.2 Sample Preparation

Commercial SiO_*x*_/C powder (HESO obtained from AMPRIUS) and nanostructured fumed Al_2_O_3_ (Evonik Operations GmbH) were used. Two surface variations of the nanoparticles were used, hydrophilic and hydrophobized. The hydrophobized surfaces were achieved by surface modification with organosilanes of the hydrophilic particles. A lab‐scale high‐energy mixer from Somakon Verfahrenstechnik UG (Somakon mixer MP‐GL) was used for the dry coating process. The mixing unit has a volume of 0.5 L and two very high‐speed rotating rotors with four blades each. The SiO_*x*_/C powder was mixed with 1 wt% of nanostructured Al_2_O_3_ powder in the high‐energy mixer. The mixing process was defined by first mixing for 1 min at 500 rpm to homogeneously mix the two powders. The key step for the process is to deagglomerate the nanostructured Al_2_O_3_, to ensure the aggregates are small enough that the Van der Waals forces combined with the mechanical mixing are sufficient for them to adhere to the surface of the active anode material (AAM). Therefore, the mixing intensity was increased to 2000 rpm during 1 min, for three times. The coating process was optimized to ensure proper deagglomeration of the nanoparticles while preventing damage to the host particles. Different time and speed settings were previously tested to determine the optimal conditions presented in this study.

### 2.3 Anode Active Material Interaction with Electrolyte

The powdered materials (SiO_*x*_/C uncoated and coated with 1 wt% of hydrophobized and hydrophilic Al_2_O_3_) were mixed with a commercially purchased electrolyte (1.0 m LiPF_6_^−^ in EC:DEC = 50:50 (v/v)) were mixed with a commercially purchased electrolyte (1.0 m LiPF6− in EC:DEC = 50:50 v) from Sigma Aldrich, simulating the powder/electrolyte ratio used during cell assembly. To study the effect of alumina as a coating, two additional samples were prepared with the uncoated material and the two grades of Al_2_O_3_ free‐flowing, instead of coated on the surface of the SiO_*x*_/C material. Additionally, to investigate the effect of Al_2_O_3_ as an electrolyte additive, two other samples were prepared with only the electrolyte and the nanoparticles—without the SiO_*x*_/C material. The samples were prepared in the glove box and stored in glass vials, sealed with a lid together with parafilm to ensure minimum oxygen and water exposure. After preparation, the samples were taken out of the glove box to be stored in a 50 °C oven for 2 weeks. The soaked powders were separated from the electrolyte inside the glove box by individual filtering using filter paper (grade 3) and pouring diethyl carbonate (DEC) solvent to remove excess electrolyte. The powder samples were then put in glass vials and dried at 50 °C under vacuum overnight.

### 2.4 Characterization

#### 2.4.1 SEM‐EDX

The particles and their morphology were investigated with a “JSM‐7600F” SEM from Jeol. The accelerating voltage was set to 1 kV, and the beam current was 30 pA. A graphite tape was used to attach the samples to the sample holders. EDX measurements were conducted with an equipped X‐Max 150 mm^2^ detector (Oxford Instruments) and processed with Aztec software. The accelerating voltage and beam current were increased to 20 kV and 500 pA, respectively. The cross sections of the electrodes were prepared by embedding them in an organic resin and cutting via microtomy.

#### 2.4.2 Surface Area Measurements (BET)

The surface area was measured according to Brunauer–Emmett–Telle (BET) theory. Three‐point BET measurements were conducted using a Micromeritics TriStar 3000 with a nitrogen/helium flow (28.6% N_2_). The samples were degassed under vacuum (≤10 μmHg) at 150 °C during 20 min before the measurement.

#### 2.4.3 Nuclear Magnetic Resonance Spectroscopy (NMR)

DMSO‐d6 was filled in an NMR tube, in which the coaxial insert was inserted. Fluorine (19F) and phosphorous‐NMR (31P) were referenced using the external standards of H_3_PO_4_ (0 ppm) and F_6_C_6_ (2164.9 ppm) in DMSO‐d6.

#### 2.4.4 *X‐Ray* Photoelectron *Spectroscopy (XPS)*

The samples were prepared in a glovebox under dry nitrogen. Before measurement, the samples were evacuated at room temperature down to 10^−8^ mbar. All measurements were carried out using a monochromatic X‐ray spot of the specimen surface. The XPS measurements were conducted on an ESCALAB 250xi system from Thermo Fisher Scientific, using an Al K α excitation source. The diameter of the measurement spot was 650 μm. The resolution of the measurements was in general 0.1 eV.

### 2.5 Electrochemical Measurements

To manufacture the electrodes, a uniform slurry was produced with active material, conductive agent and binder in deionized water with a mass ratio of 80:10:10 (wt%). The silicon‐based powder prepared by dry coating was used as the active material, Super C65 carbon black (denoted by CB, TIMCAL) and single‐walled carbon nanotubes (denoted by single‐walled carbon nanotubes (SWCNT), TUBALL BATT H_2_O 0.4%) as the conductive agents, Na‐CMC (denoted CMC by Alfa Aesar) as the binder and water as the solvent. For the electrodes containing SWCNTs, a dispersion of 0.4 wt% SWCNTs and 0.6 wt% CMC in water was added to the slurry. As the SWCNT dispersion contains a small amount of CMC, the amount of Na‐CMC was reduced concerning the electrodes without SWCNTs to maintain the same weight ratio. The electrodes were made by casting the slurries onto a copper foil using a doctor‐blading coater. After drying at room temperature for 15 min and at 80 °C for 30 min, the electrodes were transferred into a vacuum oven to dry for another 12 h at 110 °C. The active material loading was ≈2.2 mAh cm^−2^, assuming a specific capacity of 1400 mAh g^−1^. The negative electrode‐to‐positive electrode capacity ratio (N/P) was determined to be 1.3. The cells were assembled as CR2032‐type coin cells (MTI Corporation) in an argon‐filled glove box (Glovebox Systemtechnik GmbH). A solution of 1 molar LiPF_6_^−^ in ethylene carbonate (EC) and ethyl methyl carbonate (EMC) (50:50 wt:wt, Sigma‐Aldrich) was used as an electrolyte (70 μL). The counter electrode used was lithium metal (Rockwood Lithium GmbH) in half‐cells and NMC 811 in full‐cells (Nei Corporation) and Celgard 2500 was used as the separator. The NMC cathodes were dried in a vacuum oven at 120 °C for 12 h before being transferred to an argon‐filled glovebox. The cells were soaked for 12 h prior to testing. Electrochemical performances were carried out using a BioLogic battery cycler inside a Battery Test Chamber with voltage ranges of 0.05–1.0 V for half‐cell and 3.0–4.2 V for full cells. During cycling, the C rate was increased every five cycles, starting from 0.1/0.1 (charge/discharge) to 0.2/0.2, 0.5/0.5, 1.0/1.0, and 1.0/2.0 C. After, the cells were cycled at 1.0/1.0 C for an additional 125 cycles.

## 3 Results and Discussion

### 3.1 Effect of Hydrophobized Al_2_O_3_ Amount on Coating Characteristics and Electrochemical Performance

The SiO_*x*_/C material was coated with different amounts of hydrophobized Al_2_O_3_ (0.5, 1, 2, 5, and 10 wt%). To characterize the materials and understand the effect of the coating on the surface, the samples were analyzed by SEM (**Figure** **2**). The SEM images of the coated anode active material show the presence of the Al_2_O_3_ nanoparticles on the surface and the effect of the coating fraction on the coverage of the material. This material coated with 0.5 wt% of Al_2_O_3_ (Figure 2c,d) shows a very sparse coating, with most of the surface uncoated. When 1 wt% of Al_2_O_3_ is used (Figure 2e,f), the coverage with the fumed metal oxide significantly increases, achieving a homogeneous coverage, while still exposing most of the surface of the active material. When the material is coated with 2 wt% of Al_2_O_3_ (Figure 2g,h) the surface coverage increases significantly, highly decreasing the exposure of the surface of the active material. With 5 wt% of Al_2_O_3_ (Figure 2i,j), a continuous layer of metal oxide is achieved, almost completely covering the surface. Finally, for 10 wt% of Al_2_O_3_ (Figure 2k,l), a multiayer coating is achieved.

Figure 2SEM images of SiO*x*/C coated with different percentages of hydrophobized Al_2_O_3_: a,b) Uncoated; c,d) 0.5 wt%; e,f) 1 wt%; g,h) 2 wt%; i,j) 5 wt%; k,l) 10 wt%.

**Figure:**
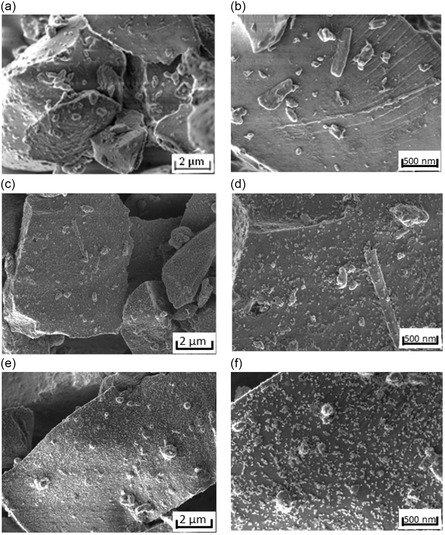

**Figure:**
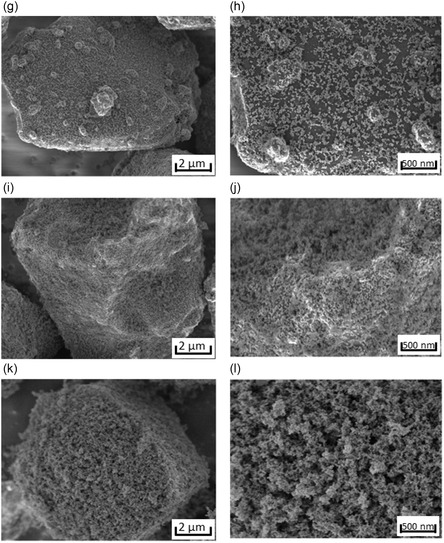

**Figure 3:**
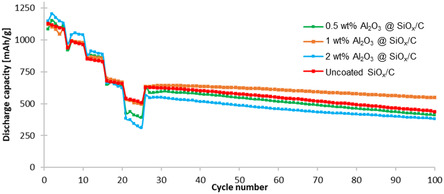
Cycling performance of SiO_*x*_/C anode uncoated, and coated with 0.5, 1 and 2 wt% of hydrophobized Al_2_O_3_ (average of three cells for each material, using NMC 811 as counter electrode).

To understand the effect of the coating amount, the surface area was measured using the BET method. The results are summarized in **Table** **1**. While the BET surface area of the pristine SiO_*x*_/C material is ≈1.1 m^2^ g^−1^, only 0.5 wt% of nanostructured aluminium oxide is enough to increase it to 1.6 m^2^ g^−1^. With increasing amounts of Al_2_O_3_ as a coating on the surface of the host material, the value increases to 1.6 m^2^ g^−1^ for 1 wt%, to 10 m^2^ g^−1^ for 10 wt% coatings. To improve electrochemical performance, it's crucial to ensure an optimal surface area of the anode active material. On one hand, with a lower surface area, the contact area with the electrolyte decreases, which is expected to decrease side reactions and hence prevent the depletion and deterioration of the electrolyte, thus improving its performance.^[^ ^53^, ^54^ ^]^ However, increasing the surface area to volume ratio has been presented as an effective way to improve the battery energy capacity per unit volume, due to the increase in efficiency of the diffusion of Li^+^ into the electrode.^[^ ^42^, ^54^, ^55^ ^]^ To examine how the amount of coating affects cycling performance, samples coated with 0.5, 1, and 2 wt% of hydrophobized Al_2_O_3_ were tested in coin cells. **Figure** **3** compares the cycling performance of these coated materials, with a voltage range of 3.0–4.2 V, to uncoated SiO_*x*_/C.

**Table 1:** BET surface area of SiO_*x*_/C coated with different amounts of alumina.

Sample	BET surface area [m^2 ^g^−1^]	Standard deviation [m^2 ^g^−1^]
SiO_*x*_/C	1.1	±0.2
0.5 wt% hydrophobized Al_2_O_3_ @ SiO_*x*_/C	1.6	±0.2
1 wt% hydrophobized Al_2_O_3_ @ SiO_*x*_/C	1.9	±0.2
2 wt% hydrophobized Al_2_O_3_ @ SiO_*x*_/C	3.0	±0.3
5 wt% hydrophobized Al_2_O_3_ @ SiO_*x*_/C	5.8	±0.3
10 wt% hydrophobized Al_2_O_3_ @ SiO_*x*_/C	10	±0.6

As depicted in Figure 3, the 1 wt% Al_2_O_3_‐coated SiO_*x*_/C anode exhibited the best capacity retention, maintaining a discharge capacity of 547 mAh g^−^^1^ with a capacity retention of 49% after 100 cycles (related to the first cycle with rate of 0.5 C). The uncoated SiO_*x*_/C reference showed a capacity of 435 mAh g^−^^1^ with a capacity retention of 36%, while the 0.5 wt% Al_2_O_3_‐coated sample achieved a capacity of 382 mAh g^−^^1^ with a capacity retention of 33%. While the 2 wt% coating initially achieved higher discharge capacities, its performance deteriorated at higher rates, displaying 409 mAh g^−^^1^ with a capacity retention of 38% after 100 cycles. The observed performance variation can be attributed to the dual role of the Al_2_O_3_ coating. At an optimal level (1 wt%), the nanostructured Al_2_O_3_ forms a protective yet permeable layer, stabilizing the solid electrolyte interphase (SEI) and mitigating side reactions during cycling. However, when the Al_2_O_3_ content exceeds this level, the excessive coating introduces additional interfacial resistance, impeding lithium‐ion transport and reducing electronic conductivity, which explains the capacity loss observed with increasing coating thickness. Conversely, a thinner coating (0.5 wt%) may be insufficient to provide these protective benefits, yet still partially obstruct lithium‐ion transport, increasing charge‐transfer resistance without adequately stabilizing the electrode. The superior performance of the 1 wt% Al_2_O_3_‐coated sample suggests that this composition achieves the optimal balance between surface protection and ionic/electronic transport, leading to improved cycling stability and capacity retention. Therefore, as a standard, further analysis was performed using 1 wt% of coating.

### 3.2 Effect of Coating Particles Wettability

**Figure 4:**
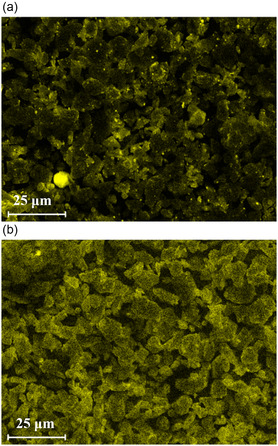
SEM EDX mapping of Al of SiO_*x*_/C coated with 1 wt% of a) hydrophilic Al_2_O_3_; b) hydrophobized Al_2_O_3_.

To investigate the influence of coating particle wettability on anode performance, the SiO_*x*_/C material was coated with 1 wt% of both hydrophilic and hydrophobized nanostructured fumed Al_2_O_3_. The elemental mapping of the coated samples gives insightful information about the effect of surface wettability on particle interaction, powder spreading, and coating quality (**Figure** **4**).

In the elemental mapping, the aluminum signal detected a homogeneous coverage of the guest particles without agglomeration for the SiO_*x*_/C dry coated with 1 wt% of hydrophobized Al_2_O_3_ (Figure 4b). Conversely, the hydrophilic nanoparticles (Figure 4a) showed much poorer spreadability than the hydrophobized material, as seen by the uneven powder coating and presence of agglomerates on the surface of the host particle. This observation could be related to the water adsorption on hydrophilic nanoparticles, which allows for the formation of capillary bridges between asperities, ultimately increasing the overall particle interactions. Capillary bridges enhance the adhesion force between two particles by roughly one order of magnitude in comparison to van der Waals forces. Thus, this large particle–particle interaction force between hydrophilic nanoparticles could lead to poor flowability and powder spreadability, often combined with low packing density.^[^ ^50^ ^]^

**Figure 5:**
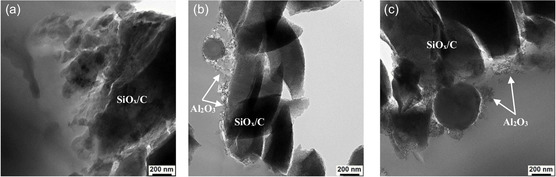
TEM images of SiO_*x*_/C a) uncoated and coated with 1 wt% of b) hydrophilic Al_2_O_3_ and c) hydrophobic Al_2_O_3_.

For a more in‐depth microstructure analysis, transmission electron (TEM) was employed to examine the cross sections of the coated SiO_*x*_/C particles. As depicted in **Figure** **5**, the coating layers exhibit an aggregated structure composed of secondary particles formed from primary particles. These aggregated structures adhere to the surface of the anode active material, creating a porous coating layer. This porosity, characterized by channels and voids, is believed to facilitate faster lithium‐ion migration by allowing the electrolyte to more easily penetrate the coating layer.^[^ ^56^ ^]^ Additionally, this could significantly reduce the impedance associated with the insulating Al_2_O_3_ coating, considering the higher ionic conductivity of liquid electrolytes compared to solid metal oxides.^[^ ^57^, ^58^, ^59^ ^]^ The porous nature of the coating layer is further corroborated by BET measurements, which reveal an increase in specific surface area with increasing coating percentage, as shown in Table 1.

### 3.3 Interaction with Electrolyte

LiPF_6_^−^ based electrolytes are widely used in commercial lithium‐ion batteries due to their high ionic conductivity and decent electrochemical stability. However, they come with significant challenges, largely because of the high hygroscopic nature of LiPF_6_^−^. This sensitivity to moisture leads to several critical issues, including high reactivity to even small amounts of water and poor thermal stability at elevated temperatures.^[^ ^60^, ^61^, ^62^ ^]^ When exposed to trace amounts of H_2_O, LiPF_6_^−^ becomes thermally unstable, decomposing into reactive and corrosive species like POF_3_, PF_5_, and HF. These by‐products can degrade the SEI layer on the electrode, damage the electrolyte solvents, and even trigger the dissolution of transition metals.^[^ ^60^, ^63^, ^64^ ^]^ In the first stage of the reaction, it is proposed that the reaction mechanism starts with the hydrolysis of LiPF_6_^−^^[^ ^63^, ^65^ ^]^

(1) $${\text{LIPF}}_{6}⇌\text{LiF}+{\text{PF}}_{5}$$

followed by the interaction between the phosphorus pentafluoride and water to form HF and POF_3_

(2) $${\text{PF}}_{5}+H_{2}O⇌{\text{POF}}_{3}+2\text{HF}$$

The electrolyte solvent EC can react with PF_5_ species, leading to ring‐opening reactions that result in polymerization into polyethylene and the formation of CO_2_ gas. Similarly, DEC reacts with HF and PF_5_, which not only produces additional HF but also generates CO_2_ gas, as illustrated by Equation (3) and (4)^[^ ^9^, ^14^ ^]^

(3) $${\text{PF}}_{5}+C_{2}H_{5}{\text{OCOOC}}_{2}H_{5}⇌C_{2}H_{5}{\text{OCOOPF}}_{4}+\text{HF}+C_{2}H_{4}$$

(4) $$\text{HF}+C_{2}H_{5}{\text{OCOOPF}}_{4}⇌{\text{PF}}_{4}\text{OH}+C_{2}H_{5}F+{\text{CO}}_{2}$$

**Figure 6:**
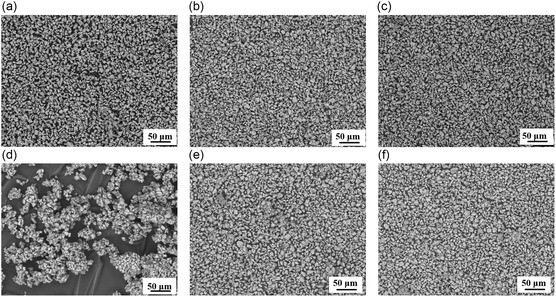
SEM images of SiO_*x*_/C a,d) uncoated and coated with 1 wt% of b,e) hydrophillic and c,f) hydrophobized Al_2_O_3_; (a–c) before and (d–f) after soaking in electrolyte.

The HF formation is considered to critically affect the battery performance, not only by causing loss of electrolyte but also by attacking the electrode surface. The biggest problem related to the anode is the Solid Electrolyte Interface (SEI) formation, which is ascribed to the interaction of the material with reactive species such as HF and PF_5_, as well as to thermal or electrochemical degradation.^[^ ^66^ ^]^ Coating layers may be used in Si‐based anodes to shield the reactive Li_*x*_Si_*y*_ phase during the alloying process from direct contact with the electrolyte.^[^ ^67^ ^]^ To understand the impacts of the coating on the surface morphology of the materials, each sample was analyzed before and after being soaked in electrolyte for 2 weeks at 50 °C. To simulate the ratio used during cell assembly, a ratio of 125 mg powder per ml electrolyte was used. The main goal of the experiment was to induce an interfacial reaction without electrochemical processes between the electrolyte and the active materials.^[^ ^39^ ^]^ High‐resolution SEM images of SiO_*x*_/C uncoated (a,d) and coated with hydrophilic (b,e) and hydrophobized (c,f) Al_2_O_3_, before (a,b,c) and after (d,e,f) soaking in the electrolyte are presented in **Figure** **6**.

The surface morphology of the pristine SiO_*x*_/C is significantly altered after being soaked in electrolyte. The SEM image (Figure 6b) reveals the agglomeration of particles on the surface. In contrast, the Al_2_O_3_ seems to protect the surface of the SiO_*x*_/C material, as both samples with coating maintained their surface morphology after interacting with the electrolyte.

To understand the effect of Al_2_O_3_ in electrolyte degradation, the content of PF_6_^−^ was used to detect the presence of with ^19^F NMR. Additionally, ^19^F NMR was also used to detect the presence of hydrofluoric acid in each sample. To study the effect of the coating, samples containing the uncoated active material and Al_2_O_3_ (both hydrophilic and hydrophobized) as coating and as an additive were analyzed. The results are summarized in **Table** **2**.

**Table 2:** HF and PF_6_^−^ content in each sample after interacting with electrolyte measured by NMR.

Compound	Mol%	
	HF	PF_6_^−^
Electrolyte reference	6.7	90.2
Uncoated SiO_*x*_/C	42.7	0.7
1 wt% hydrophilic Al_2_O_3_ @ SiO_*x*_/C	4.5	82.3
1 wt% hydrophobized Al_2_O_3_ @ SiO_*x*_/C	3.6	84.3
1 wt% hydrophobized Al_2_O_3_ + SiO_*x*_/C	39	12

The introduction of SiO_*x*_/C to the electrolyte significantly increases the HF content from 6.7% to 42.72% compared to the reference electrolyte. However, coating the AAM with 1 wt% of either hydrophilic (4.51%) or hydrophobized (3.61%) Al_2_O_3_ dramatically reduces this increase. This suggests that the uncoated SiO_*x*_/C accelerates HF formation, while the Al_2_O_3_ coating acts as both a surface protector and an HF neutralizer. In contrast, when Al_2_O_3_ is simply added to the uncoated SiO_*x*_/C material, the HF content remains high at 39%. This is slightly lower than the uncoated SiO_*x*_/C, but still significantly higher than the coated samples, which further emphasizes the importance of the Al_2_O_3_ coating in protecting the surface, as demonstrated in Figure 6. The Al_2_O_3_ coating appears to react with the detrimental HF, likely due to HF attacking the alumina. This “HF or H_2_O scavenging effect” has been previously reported in the literature^[^ ^68^, ^69^, ^70^ ^]^

(5) $$6\text{HF}+{\text{Al}}_{2}O_{3}\to 2{\text{AlF}}_{3}+3H_{2}O$$

**Figure 7:**
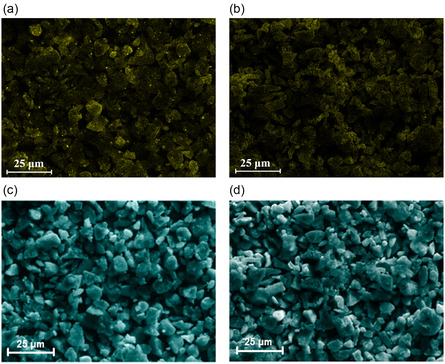
EDX images representing the distribution of Al and F on a,c) hydrophilic and b,d) hydrophobized Al_2_O_3_ after interacting with electrolyte.

By reacting with the HF, the coating layer protects the underlying anode active material. This is supported by evidence of fluorination within the oxide coatings, as showed in the elemental mapping of the coated samples (**Figure** **7**).

In addition to HF content, electrolyte degradation is a critical concern. To assess this, the presence of PF_6_^−^ was measured in each sample. The reference electrolyte contains 90.21% PF_6_^−^, but this drops drastically to 0.73% when SiO_*x*_/C is introduced, indicating significant electrolyte decomposition. In contrast, the PF_6_^−^ content increases to 82.25% and 84.2% when the SiO_*x*_/C is coated with hydrophilic and hydrophobized Al_2_O_3_, respectively. When Al_2_O_3_ is added to the electrolyte with uncoated SiO_*x*_/C, the PF_6_^−^ content is only 12%, again highlighting that the coating, rather than just the presence of Al_2_O_3_, is crucial for preserving electrolyte integrity. Maintaining a higher PF_6_^−^ content, and therefore less electrolyte decomposition, is vital for reducing gas generation within the cell, ultimately improving cell performance. The results from ^1^ ^9^F NMR clearly indicate that both hydrophilic and hydrophobized Al_2_O_3_ coatings not only neutralize HF but also help maintain the stability of the electrolyte.

### 3.4 Electrode Characterization

**Figure 8:**
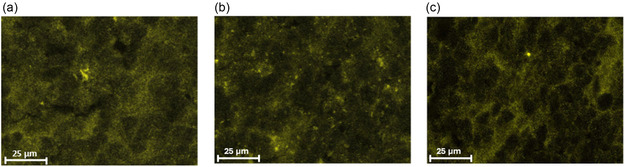
EDX‐mapping of Al of electrodes manufactured with SiO_*x*_/C coated with a) hydrophilic and b) hydrophobized Al_2_O_3_ and c) SiO_*x*_/C uncoated and hydrophilic Al_2_O_3_ added as additive.

The microstructure of the electrode is directly related to the distribution of the active material on the current collector, which makes slurry dispersibility an important parameter to consider to achieve good cell performance.^[^ ^52^, ^71^, ^72^, ^73^ ^]^ The surface of the finished electrodes, manufactured with active material coated with 1 wt% of hydrophilic and hydrophobized Al_2_O_3_, was analyzed by SEM‐EDX (**Figure** **8**a,b). Additionally, to have a further insight on the effect of using Al_2_O_3_ as a coating, electrodes using the metal oxide as an additive dispersed in the slurry were also studied (Figure 8c).

The surface of the different electrodes shows the presence of not only the active material but also the additives and the bridges obtained due to the SWCNTS, as described in Figure S1, Supporting Information. The EDX‐mapping shows that the Al_2_O_3_ is well dispersed on the surface of the electrode. However, the hydrophilic coating appears more homogeneous than the hydrophobized. When the uncoated SiO_*x*_/C and Na‐CMC are mixed during the slurry production, the hydrophilic binder is only partially adsorbed on the anode material surface due to the hydrophobized carbon shell, which makes the dispersibility of the anode slurry poor.^[^ ^73^ ^]^ When the active material is coated with the hydrophilic particles, it enhances the interaction with Na‐CMC during the slurry production process, which increases the adsorption amount of binder on the anode active material surface and improves the dispersibility of the slurry.^[^ ^73^ ^]^ When a hydrophilic binder is mixed with a hydrophobic powder, the hydrophilic molecules will preferentially interact with each other due to their similar polarity. This can result in incomplete coverage of the hydrophobic powder surface. In order to verify if the Al_2_O_3_ is still present as a coating and if the particles were not detached during the electrode manufacturing process, cross‐sections from the finished anodes were prepared and investigated by SEM‐EDX.

**Figure 9:**
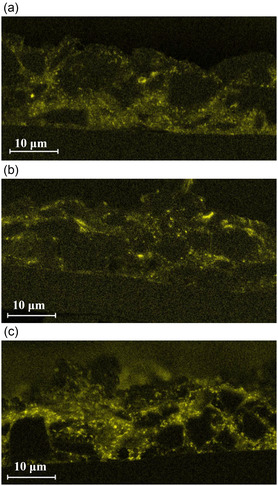
Cross‐section images of electrodes manufactured with SiO_*x*_/C coated with a) hydrophilic and b) hydrophobized Al_2_O_3_ and c) uncoated with hydrophilic Al_2_O_3_ as additive.

The image of the anode containing the fumed alumina‐coated SiO_*x*_/C particles (**Figure** **9**a,b) shows that the coating is attached to the anode's active material, indicating that the particle structure was maintained during the mixing process. Conversely, the electrodes manufactured with Al_2_O_3_ as an additive (Figure 9c) show the dispersed and detached particles in agglomerate form, which indicates that the mixing forces introduced during slurry preparation are not capable of the deagglomeration achieved with the dry particle coating technique.

### 3.5 Electrochemical Measurements

**Figure 10:**
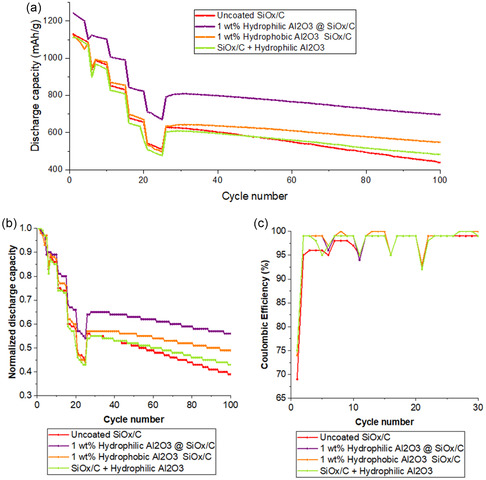
Cycling performance of SiO_*x*_/C anode uncoated, coated with 1 wt% of hydrophilic and hydrophobized Al_2_O_3_ and uncoated with hydrophilic Al_2_O_3_ as an additive (average of three cells for each material, using NMC 811 as counter electrode): a) absolute and b) normalized discharge capacities, and c) coulombic efficiency.

To investigate the impact of nanostructured fumed Al_2_O_3_, as well as their surface wettability, on battery performance, electrodes were manufactured using the three different active materials and tested in coin cells. The cycling performances and coulombic efficiencies obtained with voltage ranges of 3.0–4.2 V comparing electrodes manufactured with SiO_*x*_/C uncoated and coated with 1 wt% of hydrophilic and hydrophobized Al_2_O_3_, as well as with hydrophilic Al_2_O_3_ used as an additive, are presented in **Figure** **10**. The electrochemical parameters (charge and discharge capacity, as well as ICE), are presented in Table S1, Supporting Information.

The SiO_*x*_/C materials coated with either grade of Al_2_O_3_ show lower initial specific capacity losses than the uncoated samples, which indicates that the use of a coating layer can impact the early stages of the anode's lithiation process by preventing direct electrolyte contact with the SiO_*x*_/C surface. This results in reduced SEI formation and lower irreversible capacity loss, leading to a stable interface and superior long‐term cycling performance.

A coating layer can decrease the surface area of the anode active material, ultimately leading to a reduction in initial SEI losses.^[^ ^41^, ^74^ ^]^ While the total BET surface area of the coated powder increases due to the intrinsic porosity of Al_2_O_3_, the electrochemically active surface of the underlying SiO_*x*_/C exposed to liquid electrolyte is effectively reduced by the conformal oxide layer. This phenomenon helps suppress excessive SEI growth directly on the SiO_*x*_/C, even though the external BET measurement registers a net increase in surface area. Consequently, the coating's passivation role leads to fewer early‐cycle side reactions, as evidenced by the lower initial capacity losses (Figure 10c) and improved stability (Figure 10a).

**Figure 11:**
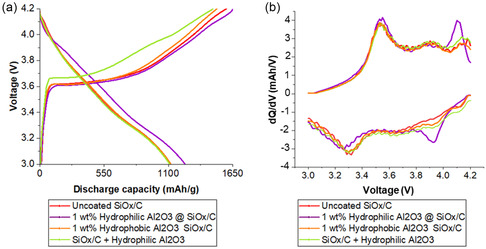
Cycling performance of SiO_*x*_/C anode uncoated, coated with 1 wt% of hydrophilic and hydrophobized Al_2_O_3_ and uncoated with hydrophilic Al_2_O_3_ as an additive (average of three cells for each material, using NMC 811 as counter electrode): a) voltage profiles for the 1st cycle and b) differential capacity curves for the 2nd cycle.

To further elucidate the role of the Al_2_O_3_ coating in SEI formation and its interaction with the electrolyte during the first cycle, the initial charge and discharge voltage profiles of the different electrodes are presented in **Figure** **11**a. The uncoated sample with the Al_2_O_3_ additive exhibits a first charge curve at higher voltages than the coated materials, indicating greater resistance to initial lithium insertion. process. Furthermore, the first discharge curve of the hydrophilic Al_2_O_3_‐coated anode occurs at a higher voltage than the others, signifying enhanced lithium extraction, which could be attributed to improved electronic conductivity within the electrode or a more favorable interaction between lithium ions and the coated SiO_*x*_/C surface. These results further reinforce the passivation role of the Al_2_O_3_ coating in stabilizing the electrode/electrolyte interface and improving lithium transport kinetics, thereby contributing to enhanced cycling performance.

The d*Q*/d*V* analysis (Figure 11b) further supports these findings by providing insights into the lithium insertion/extraction mechanisms and interfacial stability of the different anodes. The hydrophilic Al_2_O_3_‐coated electrode exhibits sharper and more distinct d*Q*/d*V* peaks, indicating lower resistance, improved lithium‐ion transport, and more stable electrochemical reactions. This superior performance is consistent with its enhanced dispersibility and uniform coverage, which helps minimize SEI buildup and maintain efficient lithium intercalation/deintercalation pathways. These results align with previous observations, confirming that the hydrophilic Al_2_O_3_ coating plays a crucial role in optimizing electrode performance by reducing lithium insertion barriers, stabilizing the SEI, and maintaining efficient lithium‐ion transport.

The use of nanostructured fumed Al_2_O_3_ as a coating is an effective method to protect the anode material surface and suppress detrimental side reactions, maintain electrolyte stability, and decrease HF formation, which overall reflects the better long‐term performance of the cells. It is apparent that electrodes containing hydrophilic nanostructured Al_2_O_3_ coating exhibited superior rate capability and cycle life compared to those with a hydrophobized coating. The enhanced performance of the hydrophilic coating can be attributed to its favorable interactions with water and the hydrophilic Na‐CMC binder during electrode fabrication, leading to improved dispersibility in the water‐based slurry. This improved dispersibility results in a more homogeneous distribution of the active material, as observed in the electrode characterization (Figure 8 and 9), which positively impacts the electrode's microstructure.^[^ ^52^, ^71^, ^72^, ^75^, ^76^ ^]^

We hypothesize that lithium ions preferentially diffuse through porous pathways within the coating layer. However, inhomogeneities in the coating distribution can force lithium ions to migrate through the coating material itself, potentially hindering their transport. In contrast, a well‐distributed coating facilitates migration through thin barriers, which is generally favored over diffusion through complex channel networks. Thus, the improved interaction between the hydrophilic coating and the binder enhances the dispersibility of Al_2_O_3_ in the slurry, compared to the hydrophobized Al_2_O_3_, potentially improving lithium diffusion and resulting in the higher initial capacity. The more inhomogeneous microstructure of electrodes with the hydrophobized coating may lead to higher tortuosity, resulting in hindered Li‐ion kinetics and ultimately, poorer electrochemical performance.^[^ ^73^, ^77^, ^78^ ^]^

The cell containing the hydrophilic Al_2_O_3_ additive in the slurry exhibits a higher discharge capacity after 100 cycles than the uncoated material but lower than the one with the coating. The advantage in comparison to the uncoated active material may be attributed to previously mentioned benefits associated with the interaction of Al_2_O_3_ with the electrolyte, such as reduced HF production and electrolyte decomposition. Nevertheless, the surface protection effect offered by the coating is absent, which explains the worse performance when compared to the cell with the material coated. Additionally, the cross‐section of the electrode (Figure 9) shows a very inhomogeneous distribution of the Al_2_O_3_ in the agglomerate state, indicating the forces introduced during slurry preparation were not sufficient for their deagglomeration.

The uncoated material results in the lowest capacity after 100 cycles, while the cell with the Al_2_O_3_ additive shows the most rapid decline in capacity. In this case, the addition of Al_2_O_3_ without the protective coating negatively impacts cell performance by introducing more inactive material, which ultimately reduces volumetric capacity, energy density, and powder density.

### 3.6 Characterization of Cycled Electrodes

**Figure 12:**
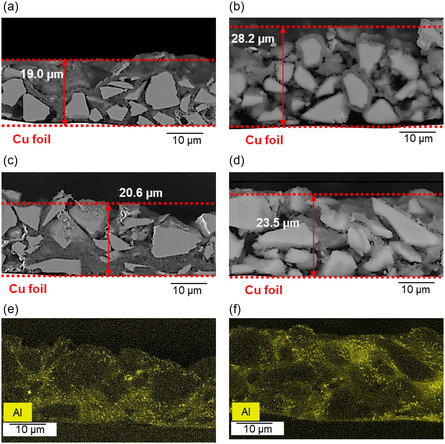
SEM‐EDX images of the cross‐sectional view of electrodes containing SiO_*x*_/C: a) uncoated, before cycling; b) uncoated, after 100 cycles; c,e) coated with 1 wt% hydrophilic Al_2_O_3_, before cycling; and d,f) coated with 1 wt% hydrophilic Al_2_O_3__3_, after 100 cycles.

Cross sectional samples of the anode were prepared and analyzed using SEM‐EDX to compare the microstructure of the best and worst performing electrodes before and after 100 cycles of cycling. The results are presented in **Figure** **12**.

Postcycling analysis of uncoated SiO_*x*_/C samples (Figure 12b) revealed significant particle pulverization compared to their precycling state (Figure 12a), particularly near the separator region. This suggests pronounced electrochemical activity and mechanical stress in these areas. In contrast, the Al_2_O_3_‐coated SiO_*x*_/C anode (Figure 12c,d), cycled under identical conditions, retained its particle integrity, demonstrating the protective effect of the coating.

To further evaluate structural changes, cross‐sectional SEM imaging was employed to assess electrode volume expansion after 100 cycles. Both electrodes exhibited substantial accumulation of the SEI layer, a passivation layer that forms on the electrode surface during initial cycling, particularly on the negative electrode. The SEI layer plays a critical role in lithium‐ion battery performance, as its composition and stability directly influence ion transport, electrode degradation, and electrolyte consumption. Consequently, SEI structure and composition are pivotal in determining battery performance, cycle life, and safety.

A layer of electrolyte decomposition products, contributing to the formation of the SEI, was observed on the surface of both electrodes postcycling. In comparison to precycling characterization, secondary components between SiO_*x*_/C particles were more apparent, likely due to electrolyte decomposition and the formation of the SEI.

Quantitative analysis revealed a significant difference in volumetric expansion between the uncoated and Al_2_O_3_‐coated SiO_*x*_/C electrodes after 100 cycles. The uncoated electrode expanded by 48%, while the Al_2_O_3_‐coated electrode exhibited a reduced expansion of 14%, suggesting superior protection against side reactions and SEI growth, as previously demonstrated by the lower amount of HF and conservation of PF_6_^−^ in the electrolyte. Moreover, a substantial portion of the uncoated electrode was composed of the SEI layer, whereas the Al_2_O_3_‐coated electrode retained a larger fraction of active material. These findings underscore the effectiveness of the hydrophilic Al_2_O_3_ coating in mitigating internal stress, preventing particle pulverization, and protecting the active material from further side reactions. Although the inherent volume change of individual SiO_*x*_/C particles is not entirely prevented by the coating, it is evident that the Al_2_O_3_ layer greatly limits extensive SEI buildup and unwanted side reactions. As a result, overall electrode thickening is reduced. Additionally, the SEM‐EDX mapping of Al confirms that the Al_2_O_3_ coating remains stable and intact on the anode particle surface after extensive cycling, demonstrating the robustness of the coating not only during the slurry preparation and anode manufacturing but also throughout the battery's cycling process.

**Figure 13:**
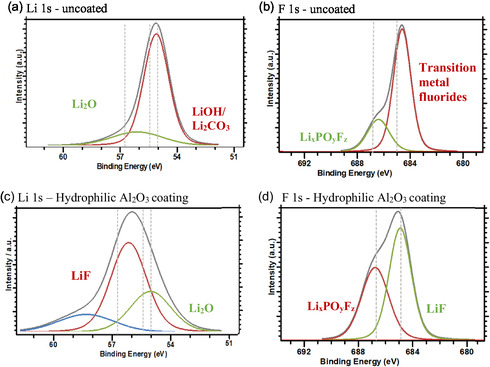
XPS analysis of a cycled electrode containing SiO_*x*_/C a,b) uncoated and c,d) coated with 1 wt% fumed Al_2_O_3:_ (a,c) Li 1s and (b,d) F 1s spectra of the surface of the electrode.

To further investigate the effect of coating on the anode surface and SEI layer, XPS spectra comparisons for the surface of uncoated and Al_2_O_3_‐coated SiO_*x*_/C anodes were discussed, as well as uncoated SiO_*x*_/C with hydrophilic Al_2_O_3_ as an additive, not as coating. The Li 1s and F 1s spectra of both electrodes are shown in **Figure** **13**.

The Li 1*s* spectra exhibit three primary peaks centered at 55, 56, and 56.6 eV. These peaks are attributed to LiOH/Li_2_CO_3_, Li_2_O/(LiF)_*x*_(LiPO_3_)_1−*x*_, and LiF, respectively.

The LiOH/Li_2_CO_3_ peak is exclusively observed in the SiO_*x*_/C anode. LiOH formation is often linked to water contamination or degradation processes.^[^ ^79^, ^80^, ^81^, ^82^ ^]^ Li_2_CO_3_, a common byproduct of electrolyte decomposition, particularly involving EC and EMC, contributes to the formation of the SEI.^[^ ^79^, ^83^, ^84^, ^85^, ^86^ ^]^ EC undergoes electrochemical reduction to form (CH_2_OCO_2_Li)_2_ and Li_2_CO_3_^[^ ^87^ ^]^

(6) $${\text{2(CH}}_{2}{\text{O)}}_{2}\text{CO}+{\text{Li}}^{+}+2e^{-}\to {\left({\text{CH}}_{2}{\text{OCO}}_{2}\text{Li}\right)}_{2}+C_{2}H_{4}$$

(7) $${({\text{CH}}_{2}\text{O)}}_{2}{\text{CO+2Li}}^{+}+2e^{-}\to {\text{Li}}_{2}{\text{CO}}_{3}+C_{2}H_{4}$$

The higher content of Li_2_CO_3_ or (CH_2_OCO_2_Li)_2_ is influenced by the EC concentration. High and low EC concentrations favor the formation of lithium carbonate and lithium ethylene dicarbonate, respectively.^[^ ^88^ ^]^ Additionally, Li_2_CO_3_ can be reduced to lithium carbide and Li_2_O, especially when using copper as a current collector.^[^ ^89^, ^90^ ^]^ While Li_2_O is predominantly an ion conductor, its low conductivity limits its practical application as an electrolyte.^[^ ^91^ ^]^

In electrolyte solutions, LiPF_6_^−^ exists in equilibrium with LiF and PF_5_, as shown in Equation (1). LiF was detected on the surface of the Al_2_O_3_‐coated, but not on the uncoated electrode. LiF's thermal and chemical stability, coupled with its ability to suppress electrode/electrolyte side reactions and enhance SEI uniformity, promotes fast lithium‐ion transport at the electrode–electrolyte interface.^[^ ^79^, ^83^, ^92^, ^93^ ^]^ Furthermore, LiF's high mechanical strength and ionic conductivity contribute to the SEI's durability. Its high shear modulus (55.14 GPa) enables it to withstand the significant volume changes associated with silicon‐based anodes.^[^ ^36^, ^87^ ^]^ LiF‐rich SEI layers have been linked to improved cycling stability and enhanced ionic transport, which directly benefits battery performance.^[^ ^94^, ^95^ ^]^

The F 1*s* core spectrum exhibits two primary peaks at around 687.0 eV and 685 eV, corresponding to the P—F bond in LiPF_6_^−^ and its decomposition products (Li_*x*_PF_*y*_/. Li_*x*_PF_*y*_O_*z*_/OPF_3−*y*_(OR)_*y*_) and LiF, respectively.^[^ ^96^ ^]^ O=P(RO)_3_ moieties are formed through the reaction of lithium alkyl carbonates (SEI species) with phosphoryl fluoride (POF_3_):^[^ ^70^, ^87^ ^]^

(8) $$3{\text{ROCO}}_{5}\text{Li}+{\text{POF}}_{2}⇌O=P{\left(\text{OR}\right)}_{3}+2\text{LiF}+3{\text{CO}}_{2}$$

While a peak is present in the F 1*s* spectrum at 684.5 eV, its shift to a lower binding energy compared to the expected LiF (≈685.0–685.5 eV) suggests that the fluorine species in this sample are not primarily LiF. This interpretation is further corroborated by the P 2*p* spectrum, which reveals a peak at 133.5 eV, corresponding to phosphates. The presence of these species, along with the absence of a characteristic LiF signal in the Li 1*s* spectrum, indicates that LiF formation in this sample is either minimal or nonexistent.

## 4 Conclusion

In this study, we explored the efficacy of dry particle coating with nanostructured fumed Al_2_O_3_ to enhance the electrochemical performance of silicon‐based anodes for lithium‐ion batteries. Our findings demonstrate significant enhancements in battery performance achieved through the application of anode active material coating with both hydrophobized and hydrophilic nanostructured fumed Al_2_O_3_. Our novel approach of dry coating SiO_*x*_/C anode materials with Al_2_O_3_ showcases a promising avenue for advancing LIB technology, particularly for Si‐based materials, while minimizing the use of inactive material. This is a practical, cost‐efficient method that can easily be transferred for other anode active materials and metal oxides as coating without the need for an additional calcination step. Surface coating with Al_2_O_3_ is an effective approach to protect the anodes surface, as well as suppress side reactions with the electrolyte, highly decrease the formation of HF and the decomposition of the electrolyte. The surface wettability of the coating particles affects the coating quality of the active material, as well as its dispersibility in the slurry and the microstructure of the finished electrode, ultimately impacting the electrochemical performance. While the hydrophobized nanoparticles deliver more homogeneous coatings on the surface of the anode active material, the hydrophilic coating enhances the wettability with binder (Na‐CMC) during the slurry production process, which improves the dispersibility in the slurry and ultimately results in a more homogeneous electrode and best overall cycling performance. By facilitating fast lithium‐ion transport and mitigating detrimental side reactions, the incorporation of hydrophilic Al_2_O_3_ presents a promising strategy for the development of high‐performance SiO_*x*_/C‐based Li‐ion batteries.

## Conflict of Interest

The authors declare no conflict of interest.

## Author Contributions

**Ana L. Azevedo Costa**: conceptualization (lead); data curation (lead); investigation (lead); methodology (lead); writing—original draft (lead). **Daniel Esken**: conceptualization (equal); supervision (lead); writing—review & editing (supporting). **Tatiana Gambaryan‐Roisman**: supervision (supporting); writing—review & editing (supporting). **Frank Menzel**: supervision (supporting); writin—review & editing (supporting).

## Supporting information

Supplementary Material
