# Supplementary material for: Dry Coating with Hydrophilic and Hydrophobized Nanostructured Fumed Alumina (Al2O3) on SiO x /C Anodes for Enhanced Lithium‐Ion Battery Performance
Source: ChemistryOpen. 2025 Apr 21;14(10):e202500170. doi: 10.1002/open.202500170 (PMC12518036; doi:10.1002/open.202500170)
Supplement: Supplementary file 1 — Supplementary Material [file OPEN-14-e202500170-s001.pdf]

## Supporting Information

*Ana Azevedo Costa, Daniel Esken, Tatiana Gambaryan-Roisman and Frank Menzel*

Si volume expansion is a critical problem for Si anodes due to material destruction, and, while carbon black (CB) is the conventional conductive additive, it cannot connect Si particles after pulverization. On the other hand, SWCNT can effectively connect Si particles and develop an electrical network of the fractured Si particles, due to the high aspect ratio of SWCNTs [1]. Studies have reported that using single-walled carbon nanotubes (SWCNTs) as conductive additives for Si-based anodes can lead to higher conductivity, as well as better rate performance and capacity retention during cycling [1–4]. Thus, herein a mixture of both conductive additives is used, to ensure the CB can improve the interface resistivity between the conductive additive and Si particles, while the SWCNT crosslinks the Si particles [1]. The SEM images presented in **Figure S 1** show the surface of the electrodes manufactured using only one (Super C65) and using both conductive additives (Super C65 and SWCNT), where the presence of carbon nanotubes are apparent.

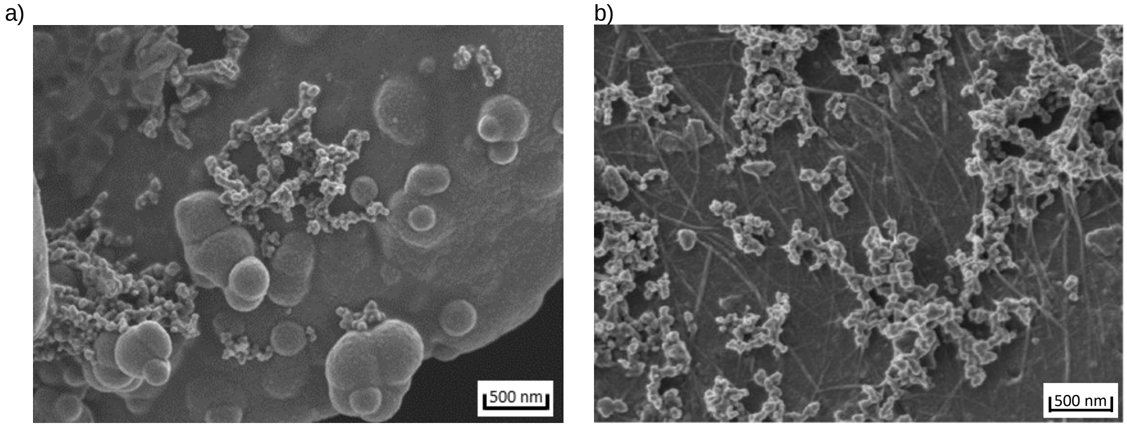


**Figure S 1** - SEM images of electrodes manufactured with $SiO_x/C$ uncoated a) with only CB and b) with CB and SWCNT.

To investigate the influence of the hybrid conductive additives on cycling performance, electrodes were manufactured with and without SWCNT and evaluated in coin half-cells. The cycling performances of both samples are presented in **Figure S 2**.

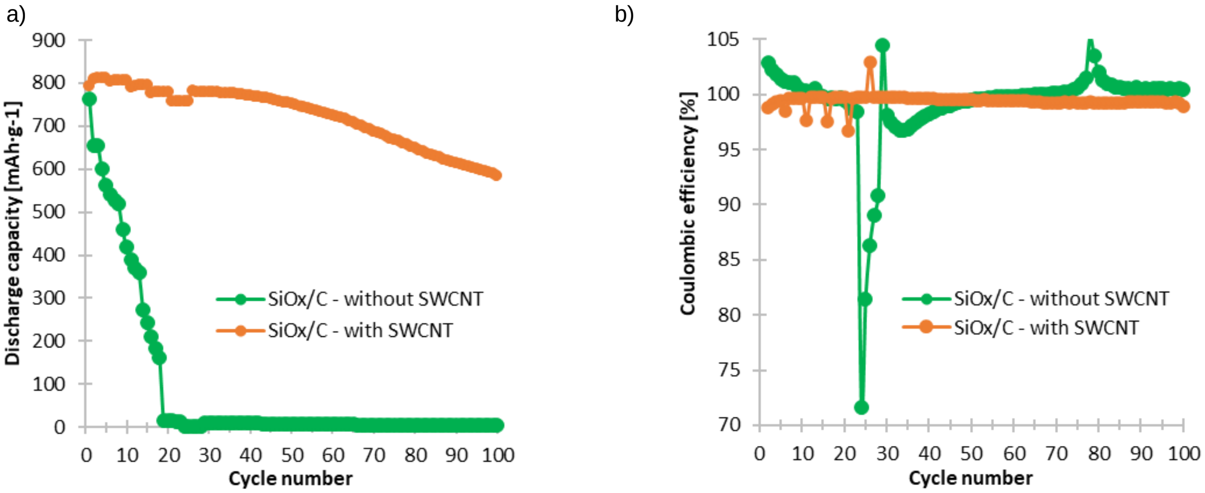


**Figure S 2** - Cycling performance of $SiO_x/C$ anode, with and without SWCNTs: a) rate capability and b) coulombic efficiency (average of three cells for each material, using lithium metal as counter-electrode).

Without SWCNT, the cell shows rapid capacity fading, dying only after around 20 cycles. Additionally, the drop in Coulombic Efficiency could be related to lithium platting. The amount of lithium ions the anode material can transport per unit of time has an upper limit. Forcing excessive current into the battery during the charging process can cause excess lithium to deposit on the surface of the electrode and form a metal layer. This phenomenon is followed by serious capacity loss. With SWCNT, there is significantly improved stability, no lithium plating, and comparably high CE in cycles 2 to 5, at C-rates up to 1 C (98.8 – 99.9%).

The normalize discharge capacity $SiO_x/C$ anode uncoated, coated with 0.5, 1 and wt% of hydrophobized $Al_2O_3$ are presented in **Figure S 3**.

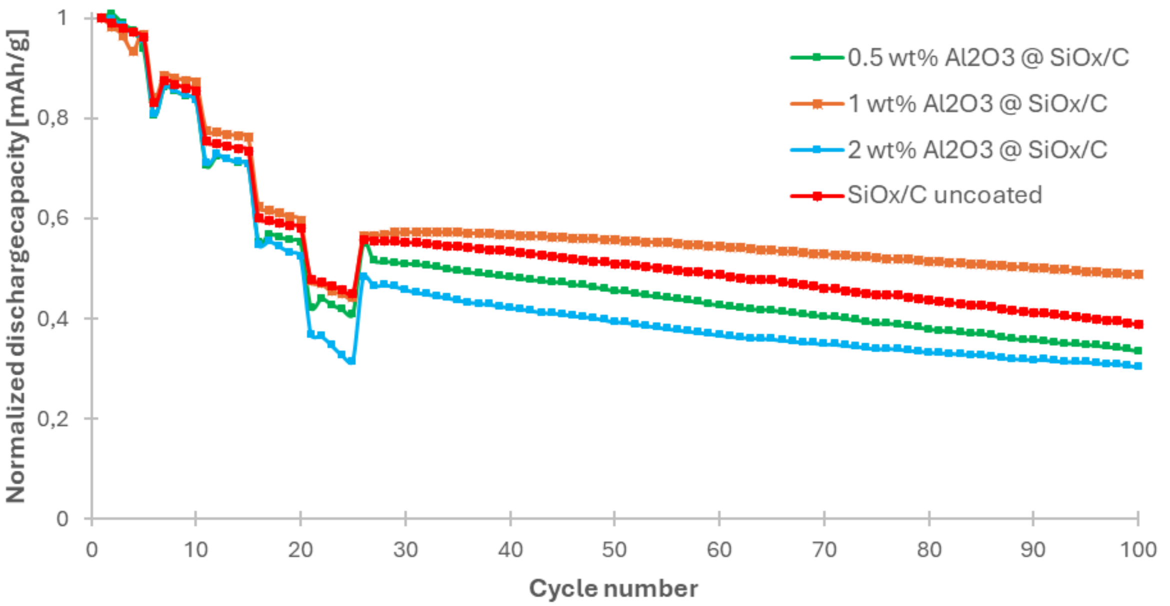


**Figure S 3** – Normalized discharge capacity of $SiO_x/C$ anode uncoated, coated with 0.5, 1 and wt% of hydrophobized $Al_2O_3$ (average of three cells for each material, using NMC 811 as counter-electrode).

**Table S1** summarizes the electrochemical performance of the electrodes, including charge and discharge capacities as well as initial Coulombic efficiency. These parameters provide insights into the impact of surface modifications on cycling stability and overall electrode performance.

**Table S1 -** Electrochemical parameters: charge capacity, discharge capacity, and initial Coulombic efficiency of the SiOx/C anodes, uncoated and

| | $SiO_x/C$ uncoated | 1 wt% Hydrophilic $Al_2O_3$ @ $SiO_x/C$ | 1 wt% Hydrophobic $Al_2O_3$ @ $SiO_x/C$ | $SiO_x/C$ + 1 wt% Hydrophilic $Al_2O_3$ |
|---|---|---|---|---|
| Charge capacity ($mAh/g^{-1}$) | 1636.16 | 1656.97 | 1515.30 | 1481.59 |
| Discharge capacity ($mAh/g^{-1}$) | 1128.95 | 1242.73 | 1121.32 | 1111.19 |
| Initial Coulombic Efficiency (%) | 69 | 75 | 74 | 75 |

**References**

[1] B.-I. Yoo, H.-M. Kim, M.-J. Choi, J.-K. Yoo, *Nanomaterials* **2022**, *12*, 3354.
[2] J. Shan, X. Yang, C. Yan, L. Chen, F. Zhao, Y. Ju, *Frontiers in Energy* **2019**, *13*, 626.
[3] X. Fan, X. Zhang, G. Hu, B. Zhang, Z. He, Y. Li, J. Zheng, *Ionics (Kiel)* **2020**, *26*, 1721.
[4] Z. Zhang, X. Han, L. Li, P. Su, W. Huang, J. Wang, J. Xu, C. Li, S. Chen, Y. Yang, *J Power Sources* **2020**, *450*, 22759.
